# Unit Response and Costs in Web Versus Face-To-Face Data Collection: Comparison of Two Cross-sectional Health Surveys

**DOI:** 10.2196/26299

**Published:** 2022-01-07

**Authors:** Elise Braekman, Stefaan Demarest, Rana Charafeddine, Sabine Drieskens, Finaba Berete, Lydia Gisle, Johan Van der Heyden, Guido Van Hal

**Affiliations:** 1 Lifestyle and chronic diseases Epidemiology and public health Sciensano Brussels Belgium; 2 Social Epidemiology and Health Policy Antwerp University Antwerp Belgium

**Keywords:** health interview surveys, data collection mode, face-to-face, web, unit response, response rate, nonresponse, data collection costs, web data, health surveys, internet penetration, web survey, costs

## Abstract

**Background:**

Potential is seen in web data collection for population health surveys due to its combined cost-effectiveness, implementation ease, and increased internet penetration. Nonetheless, web modes may lead to lower and more selective unit response than traditional modes, and this may increase bias in the measured indicators.

**Objective:**

This research assesses the unit response and costs of a web study versus face-to-face (F2F) study.

**Methods:**

Alongside the Belgian Health Interview Survey by F2F edition 2018 (BHISF2F; net sample used: 3316), a web survey (Belgian Health Interview Survey by Web [BHISWEB]; net sample used: 1010) was organized. Sociodemographic data on invited individuals was obtained from the national register and census linkages. Unit response rates considering the different sampling probabilities of both surveys were calculated. Logistic regression analyses examined the association between mode system and sociodemographic characteristics for unit nonresponse. The costs per completed web questionnaire were compared with the costs for a completed F2F questionnaire.

**Results:**

The unit response rate is lower in BHISWEB (18.0%) versus BHISF2F (43.1%). A lower response rate was observed for the web survey among all sociodemographic groups, but the difference was higher among people aged 65 years and older (15.4% vs 45.1%), lower educated people (10.9% vs 38.0%), people with a non-Belgian European nationality (11.4% vs 40.7%), people with a non-European nationality (7.2% vs 38.0%), people living alone (12.6% vs 40.5%), and people living in the Brussels-Capital (12.2% vs 41.8%) region. The sociodemographic characteristics associated with nonresponse are not the same in the 2 studies. Having another European (OR 1.60, 95% CI 1.20-2.13) or non-European nationality (OR 2.57, 95% CI 1.79-3.70) compared to a Belgian nationality and living in the Brussels-Capital (OR 1.72, 95% CI 1.41-2.10) or Walloon (OR 1.47, 95% CI 1.15-1.87) regions compared to the Flemish region are associated with a higher nonresponse only in the BHISWEB study. In BHISF2F, younger people (OR 1.31, 95% CI 1.11-1.54) are more likely to be nonrespondents than older people, and this was not the case in BHISWEB. In both studies, lower educated people have a higher probability of being nonrespondent, but this effect is more pronounced in BHISWEB (low vs high education level: Web, OR 2.71, 95% CI 2.21-3.39 and F2F OR 1.70, 95% CI 1.48-1.95). The BHISWEB study had a considerable advantage; the cost per completed questionnaire was almost 3 times lower (€41 [US $48]) compared with F2F data collection (€111 [US $131]).

**Conclusions:**

The F2F unit response rate was generally higher, yet for certain groups the difference between web and F2F was more limited. Web data collection has a considerable cost advantage. It is therefore worth experimenting with adaptive mixed-mode designs to optimize financial resources without increasing selection bias (eg, only inviting sociodemographic groups who are keener to participate online for web surveys while continuing to focus on increasing F2F response rates for other groups).

## Introduction

General population health surveys are an important data source for monitoring the health of the population and for policy making. In this regard, the Belgian Health Interview Survey (BHIS) provides periodic statistics on the health status, health care use, and health determinants of the country’s population [1]. Through the BHIS, the statistics requested by the European Statistical Office (Eurostat) in the framework of the European Health Interview Survey (EHIS) are collected. Since its inception in 1997, BHIS data collection has been undertaken through face-to-face (F2F) interviews at participants’ homes. In addition, participants aged 15 years and older are asked to fill out a paper-and-pencil questionnaire covering the most sensitive topics. This mix of the interview and self-administered modes is used to exploit the advantages of each [2]. F2F interviewing is particularly suited for the rather long and complex BHIS questionnaire [3,4], and the paper-and-pencil questionnaire reduces the risk of social desirability bias for sensitive topics and enhances privacy [2].

High internet penetration rates and widespread adaptation of the general public to the internet have encouraged experimentation with online HISs in developed countries to exploit the advantages of the web mode. Sending emails is the most cost-effective recruitment strategy for web surveys [5]. Even when using postal mail instead of email invitations, however, a web survey may have a considerable cost advantage over an F2F survey [6]. Moreover, web data collection shortens the duration of data collection [6,7] and is less demanding from a logistical point of view (eg, no interviewer training, no intensive interviewer follow-up) [4] compared to F2F data collection.

Unlike an F2F survey with a paper-and-pencil self-administered part, a web survey is completely self-administered, and this also provides advantages [5]. For example, the burden on respondents is expected to be lower when using the web mode since respondents can complete the questionnaire at a time convenient for them, possibly even spread over several time points, and no interviewer appointment is required [6]. While interviewers can guide and motivate respondents to complete the questionnaire optimally, their presence in itself (ie, social desirability bias), their characteristics (eg, age, gender, and ethnicity), and their interview strategies (eg, incorrect reading of the questions or inadequate probing) can affect the respondent’s answers [3,8].

In spite of these advantages, the web mode is rarely used as a single mode of data collection for population-based HISs due to expected noncoverage and nonresponse issues. Although the internet access rate has increased substantially over time in Europe, noncoverage remains an issue; women, older people, and lower educated people still have lower access rates [9]. Moreover, according to a recent study internet users have a better subjective health status than internet nonusers and weighting for sociodemographic characteristics does not eliminate this observed health difference [10]. In contrast, F2F data collection is expected to be the most reliable approach to obtain a nationally representative sample [11]. In theory, any household in the country can be accessed by an interviewer, and (internet) illiterate people are not excluded by default.

Regardless of the data collection mode, HIS unit response rates have been decreasing over the past decades [12-15]. Yet several meta-analyses found that web surveys perform even worse in terms of unit response rates than surveys conducted using other modes of data collection [16-19]. The higher unit response rates in F2F surveys can be attributed to the higher perceived survey legitimacy and to the persuasiveness of having someone at the doorstep [11]. More equal participation across all sociodemographic groups is also expected when using an F2F mode than when using a web mode. Laaksonen and Heiskanen [20] reported a considerably lower unit response rate of a web compared to F2F study organized in the Finnish general population (25% vs 50%, respectively). Moreover, they showed that being older, being a nonnative Finnish speaker, and being lower educated were associated with a lower probability of responding to a web survey, whereas these sociodemographic variables were not predictors of a lower response in their F2F survey.

Such direct comparisons between F2F and web surveys based on probability samples are rare. Web data collection (using postal mail recruitment) is more frequently compared to paper-and-pencil data collection in terms of coverage and unit nonresponse rates [21-25]. The aim of this study is to compare F2F data collection with web data collection using postal mail recruitment in terms of unit response and costs of a general population health survey using a sample drawn from a national population register. An additional objective is the assessment of the sociodemographic characteristics associated with unit nonresponse using the 2 modes. The hypothesis is that in 2018, our study period, the unit response rate on the web will still be lower than the F2F unit response rate, although this difference between unit response rates will probably vary among the various sociodemographic groups in the population. It is also expected that the costs will be higher in the F2F compared to the web study.

## Methods

### Study Design

Alongside the traditional F2F survey (ie, the Belgian Health Interview Survey by F2F edition 2018 [BHISF2F]), a web-based survey (ie, the Belgian Health Interview Survey by Web [BHISWEB]) was organized. For both studies, authorizations were received from the Belgian privacy commission and from the ethics committee of the Ghent University Hospital.

#### Belgian Health Interview Survey by F2F

A cross-sectional F2F study was conducted in Belgium with a target net sample size of 11,300. The survey was organized at household level, and households were selected based on the national population register, using a multistage clustered sampling procedure. For every selected household, 3 replacement households matched on statistical sector (ie, a subdivision of a municipality), household size, and age of the reference person were also selected. In addition to this cluster, a substitute cluster of 4 households with no matched characteristics to the first cluster was created in case of nonparticipation of all first cluster households. Sample substitution was applied during data collection: nonparticipating households were substituted, if necessary several times, by replacement households.

The households selected for the BHISF2F received a postal advance letter stating that an interviewer would visit and containing information on the BHIS. In households with a maximum of 4 members, all members were asked to participate. For households with at least 5 members, only 4 members (selected according to a systematic approach) were asked to participate.

The gross of the BHISF2F questions were administered through a computer-assisted personal interview (CAPI), but questions on the most sensitive topics were included in a paper-and-pencil self-administered questionnaire. The latter was completed by (nonproxy) respondents aged 15 years and older during the interview session. The questionnaires were available in Dutch, French, German, and English.

Data collection took place from January 2018 to January 2019 and was organized in collaboration with our fieldwork partner, the Belgian Statistical Office (Statbel). More information can be found in Multimedia Appendix 1 and in the methodological report of the BHISF2F [26].

#### Belgian Health Interview Survey by Web

A cross-sectional web study was conducted in Belgium with a target net sample size of 1000, a number that was feasible based on the budget set aside for this study. The national population register was used as the sampling frame. Unlike the BHISF2F, the survey was organized at the individual level and only individuals aged 16 to 85 years were selected. Moreover, individuals living in collective or institutional households and individuals living in the German-speaking region of Belgium (East Belgium, <1% of the Belgian population) were excluded from the sampling frame. A multistage clustered sampling procedure, similar to the BHISF2F, was used to select individuals. For every selected individual, 9 replacement individuals who were comparable in terms of statistical sector, sex, and age were also selected. Matched sample substitution was applied during data collection: nonrespondents were replaced, if needed several times, by replacement individuals.

Selected individuals were invited through a postal letter, and one reminder letter was sent after 7 days. The access period of the web survey was 14 days. Participants received a €10 (US $12) conditional incentive in the form of a gift voucher. The BHISWEB questionnaire was shorter than the BHISF2F questionnaire as the latter contained not only the EHIS questions but also some additional questions for national purposes. The EHIS questions corresponded with all variables requested by Eurostat in the context of EHIS wave 3 [27]. The questionnaire was available in Dutch, French, and English.

Data collection was organized together with Statbel and took place from April to November 2018, with a break during July and August. More information on the design choices made in this study can be found in Multimedia Appendix 1, Multimedia Appendix 2, and elsewhere [28].

The BHISWEB and BHISF2F studies were based on 2 mutually exclusive samples. As was made clear in the earlier descriptions, the data collection mode as well as other related design features varied between the 2 surveys. Unit response rates were therefore compared between studies using different mode systems, rather than between studies using solely different modes. To assess the differences in these unit response rates, the gross samples of the BHISF2F and the BHISWEB must be made comparable. To improve the comparability the following steps were performed: (1) only individuals invited for the BHISF2F aged 16 to 85 years and not living in an institutionalized environment or in East Belgium were included; (2) only BHISF2F individuals invited for participation between April and November 2018 (excluding the holiday months) were included to correspond with the time frame of the BHISWEB; (3) a system of weighting to adjust for the differential sample selection used in the 2 studies (gross sample for BHISF2F was n=7698 and gross sample for BHISWEB was n=6183) and consequently to adjust for the differences in their age, sex, and region distribution was applied. Weights were assigned to the people invited to the BHISWEB to make this gross sample comparable to the BHISF2F gross sample in terms of the age, sex, and region distribution. The calculation of these weights was based on cross classified data on the BHISF2F gross sample in terms of age (16-40 years, 41-65 years, and 66 years and older), sex, and region (Flemish, Brussels-Capital, and Walloon regions). The people invited to the BHISF2F all received a weight of 1.

### Analyses

First, unit response rates were calculated using the weights as obtained via the method described earlier. For the BHISWEB, the unit response rate was the number of invited individuals having completed the first questions of the 3 first modules (ie, a set of questions related to the same topic) divided by the number of all invited individuals. This web response rate did not allow noncoverage due to having no internet or computer access to be disentangled from actual nonresponse. For the BHISF2F, the individual response rate and not the household response rate was calculated. More specifically, it was the number of selected individuals from the invited households who completed the first questions of the first 3 CAPI modules divided by the number of all selected individuals from the invited households. For these calculations, we did not differentiate between responses from primary selected individuals and responses from substitutes. We looked at the number of respondents in relation to the number of invited individuals (these individuals may be primary selected or substitutes). In addition to the response rate, the response rate ratio (ie, F2F unit response rate/web unit response rate) was calculated.

Demographic variables derived from the national register (sex, age group, number of household members, region of residence, nationality, and urbanization rate) were used to calculate group-specific unit response rates for the 2 studies and response rate ratios. In addition, unit response rates and response rate ratios by level of education were calculated. Since the national register does not include information on the socioeconomic status of the invited individuals, data on the education level of invited BHISWEB and BHISF2F individuals were derived from a linkage with the Administrative Census 2011. The highest education level achieved was available in 7 categories according to the International Classification of Education and was recoded in 3 categories: low educational attainment (lower secondary education or less), intermediate educational attainment (higher secondary education and postsecondary nonhigher education), and high educational attainment (higher education). High levels of item-missingness were found for educational level (ie, this information was missing for 25.5% of all selected people). In order to get an idea of how the unit response varied with the substitution process, unit response rates per substitution wave are also presented in Table 1: wave 1 concerns the unit response rate among the initially selected individuals; waves 2 to 4 and higher concern the unit response rate among the activated substitutes in each wave.

Second, logistic regression modeling was used to study the association between unit nonresponse and mode system, sociodemographic characteristics (sex, age group, education level, number of household members, region of residence, nationality, and urbanization rate), and substitution wave (basic model).

Third, to assess whether the effect of the sociodemographic characteristics on nonresponse depended on the mode system (effect modification), interaction terms were added to the basic logistic regression model. If the interaction term was significant, we stratified the regression analyses to calculate the effect of the sociodemographic characteristics by mode system (stratified models).

Due to the high level of item-missingness on the educational level variable, regression-based multiple imputation (m=20) procedures were applied for the nonresponse analyses, presuming missingness at random. The SAS PROC MIANALYZE procedure was used for the multiple imputation. All analyses were conducted in SAS Enterprise Guide 7.1 (SAS Institute Inc). They were weighted and took into account the complex sampling designs (stratification in both studies and clustering at household level for the BHISF2F).

Last, a cost analysis was performed. The costs of the BHISWEB study were compared with the costs of an F2F study. A distinction was made between fixed costs (ie, costs regardless of the number of invitations) and variable costs (ie, costs depending on the number of invitations). The reported costs of the BHISWEB study corresponded exactly with expenditure related to BHISWEB. The costs for the F2F data collection could not be based entirely on the BHISF2F expenditure due to differences regarding the target sample size (eg, BHISF2F 11,300 vs BHISWEB 1000) and due to the specific BHISF2F financing, which included reduced tariffs from the printing company and fieldwork partner. Therefore, a cost estimation was performed for an F2F study with a target sample size of 1000 under financial conditions comparable to those in the BHISWEB study. In sum, this F2F data collection consisted of a postal advance letter and a collaboration with interviewers to contact and interview the selected individuals at home (via a CAPI including a paper-and-pencil self-administered questionnaire). Due to the higher perceived legitimacy and the persuasiveness of having someone on the doorstep, an incentive was not provided. This study was implemented with the same fieldwork partner as the BHISWEB survey. Therefore, the cost assessment for the F2F study was mainly based on their tender regarding fieldwork logistics, provision of fieldwork materials, and payment for the interviewers. Concretely, the following costs were included in the comparison: project management, information and communications technology, data warehousing, licenses for survey development, incentives, printing, packaging, postage, and interviewers.

**Table 1:** Weighted unit response rates of the Belgian Health Interview Survey by Web and the Belgian Health Interview Survey by F2F edition 2018.

Characteristics			BHISWEB^a^ (n=6183), %		BHISF2F^b^ (n=7698), %		Ratio^c^ (F2F/web)
**Sex**							
	Male	17.8		41.9		2.35	
	Female	18.2		44.3		2.43	
**Age (years)**							
	16-40	18.1		39.9		2.20	
	41-65	19.1		44.9		2.35	
	65+	15.4		45.1		2.93	
**Education**							
	Low	10.9		38.0		3.49	
	Middle	20.8		44.1		2.12	
	High	29.2		52.7		1.80	
**Nationality**							
	Belgian	19.7		43.7		2.22	
	European	11.4		40.7		3.57	
	Non-European	7.2		38.0		5.28	
**Household size**							
	1	12.6		40.5		3.21	
	2	19.3		44.3		2.30	
	3	17.9		42.0		2.35	
	≥4	20.3		44.2		2.18	
**Region**							
	Flemish	22.2		41.6		1.87	
	Brussels-Capital	12.2		41.8		3.43	
	Walloon	17.9		45.3		2.53	
**Urbanicity**							
	Urban	20.9		39.6		1.89	
	Suburban	18.9		46.3		2.45	
	Rural	21.3		53.9		2.53	
	Brussels-Capital	12.2		41.8		3.43	
**Substitution wave**							
	1	20.3		45.2		2.23	
	2	17.1		41.7		2.44	
	3	19.4		37.9		1.95	
	4	17.4		43.9		2.52	
	≥5	15.3		44.0		2.88	

^a^BHISWEB: Belgian Health Interview Survey by Web.

^b^BHISF2F: Belgian Health Interview Survey by F2F edition 2018.

^c^Ratio: face-to-face response rate/web response rate.

## Results

In total, 16.3% (1010/6183) of invited individuals participated in the BHISWEB study and 43.1% (3316/7698) of invited individuals participated in the BHISF2F study. This resulted in weighted unit response rates of 18.0% for the BHISWEB study and 43.1% for the BHISF2F study (unweighted response rates: BHISWEB 16.3%; BHISF2F 43.1%). The BHISF2F response rate was 2.39 times higher than the BHISWEB response rate. An overview of the unit response rates by sociodemographic characteristics and substitution wave is provided in Table 1. Regardless of the sociodemographic subgroup in the population, the unit response rate was higher in the F2F study compared to the web study (ratio [F2F/web]>1). Nevertheless, the extent of this difference varied between sociodemographic subgroups. Especially for people aged 65 years and older, with low education levels, of non-Belgian nationality, living in a single-person household, or living in the Brussels-Capital region, the unit response rates were higher in the BHISF2F than in the BHISWEB study (ratio [F2F/web]>2.9). In both the BHISWEB and BHISF2F studies, unit response rates were highest among the initially selected individuals (20.3% in BHISWEB and 45.2% in BHISF2F) and not among the substitutes.

Logistic regression analysis showed that unit nonresponse was significantly higher in the web study compared to the F2F study (OR 3.53, 95% CI 3.18-3.91; Table 2: basic model). The basic model also showed that some sociodemographic characteristics (ie, education level, nationality, household size, urbanicity) were significantly associated with nonresponse.

In addition, the model including interaction terms showed both significant and nonsignificant interaction terms between mode system and sociodemographic characteristics. For sex, household size, and urbanicity, no significant interaction effects were found, which means that the association between nonresponse and these characteristics did not differ by mode system. People living with 2 (OR 0.80, 95% CI 0.70-0.90) or at least 4 other household members (OR 0.78, 95% CI 0.69-0.90) were less likely to be nonrespondents than singles, as were people living in rural (OR 0.61, 95% CI 0.49-0.77) or suburban (OR 0.78, 95% CI 0.68-0.90) areas compared to people living in urban areas (ORs based on full model).

Significant interaction terms were found for age, education level, region, and nationality. Stratified models indicating the results by mode system showed that after adjusting for all relevant covariates, there was an age effect in the F2F survey; younger people were more likely to be nonrespondents (OR 1.31, 95% CI 1.11-1.54) than older people, while no age effect was found in the web survey. The stratified analyses also showed a stronger education effect in the web study compared to the F2F study (web OR low vs high education level 2.71, 95% CI 2.21-3.39; F2F OR low vs high education level 1.70, 95% CI 1.48-1.95/web OR middle vs high education level 1.63, 95% CI 1.33-1.98; F2F OR middle vs high education level 1.44, 95% CI 1.25-1.65). Moreover, the nationality of invited people was associated with responding to a web survey but not to an F2F survey. People with another European (OR 1.60, 95% CI 1.20-2.13) or a non-European (OR 2.57, 95% CI 1.79-3.70) nationality were less likely to participate in the web survey than people with Belgian nationality. Last, a region effect was found in the web survey but not in the F2F survey. People living in the Brussels-Capital (OR 1.72, 95% CI 1.41-2.10) or Walloon (OR 1.47, 95% CI 1.15-1.87) region were less likely to participate in the web survey than people living in the Flemish Region.

Both fixed and variable costs were considerably lower for web than for F2F data collection (Table 3). The total cost per completed questionnaire was almost 3 times lower for web data collection (€41 [US $48]) compared to the F2F data collection (€111 [US $131]; Table 3). Two factors accounted for most of this cost difference: payment of the interviewers (for their completed interviews, training sessions attended, and transportation costs) and more expensive project management. The fieldwork follow-up in a web study is quite straightforward and based on automatic programming, but interviewers need extensive individual follow-up. Furthermore, a project manager is paid to deliver interviewer training and perform data checking and cleaning, which is more labor extensive in an F2F study than in a web study.

**Table 2:** Results of the nonresponse analyses (outcome=nonresponse), Belgian Health Interview Survey by Web, Belgian Health Interview Survey by F2F edition 2018.

				Global models						Stratified models			
				Basic^a^, OR^b^ (95% CI)			Full^c^, OR (95% CI)			BHISWEB^d^, OR (95% CI)			BHISF2F^e^, OR (95% CI)
**Mode system (ref^f^ F2F)**													
	Web			3.53 (3.18-3.91)		N/A^g^			N/A			N/A	
**Sociodemographic characteristics**													
	**Sex (ref male)**												
		Female	0.95 (0.89-1.02)		0.95 (0.89-1.02)			N/A			N/A		
	**Age (years; ref 65+)**												
		16-40	1.12 (0.98-1.28)		N/A			0.86 (0.68-1.09)			1.31 (1.11-1.54)		
		41-65	1.02 (0.91-1.16)		N/A			0.90 (0.72-1.12)			1.11 (0.95-1.30)		
	**Education (ref high)**												
		Low	2.00 (1.79-2.24)		N/A			2.71 (2.21-3.39)			1.70 (1.48-1.95)		
		Middle	1.51 (1.34-1.70)		N/A			1.63 (1.33-1.98)			1.44 (1.25-1.65)		
	**Nationality (ref Belgian)**												
		European	1.22 (1.04-1.43)		N/A			1.60 (1.20-2.13)			1.08 (0.89-1.32)		
		Non-European	1.45 (1.20-1.75)		N/A			2.57 (1.79-3.70)			1.10 (0.86-1.41)		
	**Household size (ref 1)**												
		2	0.80 (0.71-0.90)		0.80 (0.70-0.90)			N/A			N/A		
		3	0.86 (0.74-1.01)		0.87 (0.75-1.02)			N/A			N/A		
		≥4	0.79 (0.69-0.91)		0.78 (0.69-0.90)			N/A			N/A		
	**Region (ref Flemish)**												
		Brussels-Capital	1.14 (0.99-1.31)		N/A			1.72 (1.41-2.10)			0.87 (0.72-1.05)		
		Walloon	1.09 (0.96-1.25)		N/A			1.47 (1.15-1.87)			0.93 (0.79-1.09)		
	**Urbanicity (ref urban)**												
		Rural	0.65 (0.52-0.81)		0.61 (0.49-0.77)			N/A			N/A		
		Suburban	0.85 (0.75-0.97)		0.78 (0.68-0.90)			N/A			N/A		
**Substitution wave (ref 1)**													
	2			1.16 (1.02-1.33)		1.16 (1.02-1.32)			N/A			N/A	
	3			1.20 (1.03-1.40)		1.19 (1.02-1.38)			N/A			N/A	
	4			1.10 (0.93-1.31)		1.09 (0.92-1.30)			N/A			N/A	
	≥5			1.15 (0.98-1.36)		1.04 (0.88-1.24)			N/A			N/A	

^a^Basic model: ORs based on logistic regression model with nonresponse as outcome and mode system and sociodemographic characteristics and substitution wave as independent variables.

^b^OR: odds ratio.

^c^Full model: ORs based on logistic regression model with nonresponse as outcome and mode system and sociodemographic characteristics, substitution wave, and significant interaction terms between mode system and sociodemographic characteristics as independent variables.

^d^BHISWEB (Belgian Health Interview Survey by Web): ORs based on logistic regression model with nonresponse as outcome and sociodemographic characteristics as independent variable for the BHISWEB study to show the stratified results for sociodemographic characteristics with significant interaction terms in the full model.

^e^BHISF2F (Belgian Health Interview Survey by F2F edition 2018): ORs based on logistic regression model with nonresponse as outcome and sociodemographic characteristics as independent variable for the BHISF2F study to show the stratified results for sociodemographic characteristics with significant interaction terms in the full model.

^f^ref: reference.

^g^N/A: not applicable.

**Table 3:** Cost figures^a^ for web versus F2F data collection with a target sample size of 1000, Belgian Health Interview Survey by Web and F2F mode system.

					BHISWEB^b^ (n=1010), €		F2F^c^ mode system (n=1000), €
**Fixed costs**							
	**Fieldwork logistics**						
		Project management^d^	1641			35,040	
		ICT^e^	3282			3282	
		Data ware housing	4922			4922	
		Sampling	1641			1641	
	**Licenses^f^**						
		CAPI^g^ software	N/A^h^			5375	
		Web survey software	8740			N/A	
	Total fixed costs			20,226			50,260
	Fixed costs per completed questionnaire			20			50
**Variable costs**							
	Incentive payments			10,212			N/A
	**Printing, packaging and postage**						
		Invitation letter (+folder)	5387			N/A	
		Reminder letter	4829			N/A	
		Incentive letter	880			N/A	
		Interviewer materials and advance letter (+folder)	N/A			4391	
	Interviewer laptops			N/A			5200
	Interviewer payments			N/A			51,057
	Total variable costs			21,308			60,648
	Variable costs per completed questionnaire			21			61
**Total costs**							
	Total fixed and variable costs			41,534			110,908
	Costs per completed questionnaire			41			111

^a^Costs do not include salaries for researchers.

^b^BHISWEB: Belgian Health Interview Survey by Web.

^c^F2F: face-to-face.

^d^Project management includes costs for data control, follow-up, and training of interviewers; testing the programs; and salaries of project managers and administrative employees.

^e^Information and communication technology (ICT) includes the costs for developing an ICT infrastructure to organize the fieldwork.

^f^Costs associated with the training courses for developing the computer-assisted personal interview (CAPI) and web questionnaires were not considered since these development skills had already been acquired.

^g^CAPI: computer-assisted personal interview.

^h^N/A: not applicable.

## Discussion

### Principal Findings

In the context of the BHIS, web and F2F data collection were compared in terms of unit response, taking into account the different sociodemographic groups in the population and financial costs.

A response rate of 18.0% was obtained in the BHISWEB study and 43.1% in the BHISF2F study, making the web survey response almost 2.5 times lower compared to the F2F survey response. For all sociodemographic subgroups in the population, the unit response rate was higher in the F2F study compared to the web study. Nevertheless, the difference between web and F2F was more pronounced among people aged 65 years and older, with low education level, of non-Belgian nationality, living in a single-person household, and living in the Brussels-Capital region. When taking into account only the individuals initially invited (not the substitutes of nonrespondents), the response rates were higher (20.3% in BHISWEB and 45.2% in BHISF2F). This can be explained by the fact that substitutes of hard-to-reach individuals are selected because they have similar sociodemographic characteristics and, consequently, they also have a higher chance on nonresponse [29].

Different sociodemographic characteristics were associated with nonresponding (or, conversely, with responding) in the BHISWEB compared to the BHISF2F survey. Having a non-Belgian nationality and living in the Brussels-Capital or Walloon regions were associated with a higher nonresponse rate in our web survey but not in our F2F survey. Age, on the other hand, was associated with nonresponse in the F2F study but not in the web study. In the F2F study, older people were less likely to be nonrespondents. In both studies, people with low or intermediate educational levels were less likely to respond than people with high educational levels, but this effect was stronger in the web study. The association between household size and urbanicity and nonresponse did not differ between the studies. Singles and people living in urban areas were less likely to respond to both studies.

The BHISWEB study has a considerable cost advantage over the F2F study; the total cost per completed questionnaire was almost 3 times lower (€41 [US $48]) compared to the F2F data collection (€111 [US $131]).

### Strengths and Limitations

A positive aspect of this study is the comparison of an F2F study and a web study using 2 random samples drawn from the national population register and the large amount of sociodemographic information available from both participating and nonparticipating sample members. The latter is not only due to the information obtained through the national register but also due to the efforts made by linking to the Administrative Census 2011 to obtain educational information. The use of this data is not perfect since there is a time delay of 7 years between our data collection and the last administrative census and it includes a considerable number of missing values regarding the highest level of educational attainment achieved. In order to address this item-missingness, multiple imputation procedures were applied under the missingness at random assumption.

One limitation is the fact that noncoverage due to having no internet or computer access could not be disentangled from actual nonresponse in our web survey. In Belgium, 87% of households with at least one household member aged between 16 and 74 years had access to the internet at the time of the study [30], so part of the unit nonresponse is in fact linked to noncoverage. A second limitation was the strict focus on unit response rates (and unit response rate differences between sociodemographic subgroups), although there is evidence that low response rates do not necessarily lead to large nonresponse bias (ie, the difference between the expected estimate based on the respondents and the true value in the population) [31]. A Danish interview-administered study also found that although the nonresponse rate was higher among people with low socioeconomic status, no significant association was found between health status and nonresponse [32]. Proxy measures for health were used in this study: register data on hospital admission costs and dispensed prescription medicine costs. Moreover, increasing fieldwork efforts might increase the response rate, but this is not necessarily a cost-effective way of minimizing survey error [33]. Next to unit response, assessing factors related to questionnaire breakoff and item response would be of interest, as these are less commonly studied [34].

### Comparison With Prior Work

The response rate difference of 26 percentage points between F2F versus web was higher than the mean difference of 12 percentage points reported in the recent meta-analyses of Daikeler et al [19], who compared response rates between the web and other survey modes. Nonetheless, our web survey was organized in the general population among newly recruited individuals (no panel members), and postal mail instead of email invitations were used. These factors are known to contribute to a higher response rate difference between web surveys and surveys organized using other modes [19]. For all sociodemographic groups, a higher response was found in the F2F study versus web study, which indicates that F2F data collection is still the most appropriate way to achieve acceptable response rates among all sociodemographic groups [7].

In addition, we found that the association between sociodemographic characteristics and nonresponse varied between the BHISWEB and BHISF2F surveys. In line with the results of other studies [6,20], we found, for example, that people of non-Belgian origin participate less in web surveys. This can be explained by their lower internet access rates [35-37]. Moreover, a web questionnaire is self-administered, which means that respondents should be not only internet literate but also capable of reading and fully understanding questions in the official national language. This is not the case when using F2F data collection; respondents must only be able to understand the questions posed by the interviewers, but these interviewers can clarify and repeat questions when needed. Moreover, interviewers can motivate people who are less fluent in the official national language to participate by highlighting the importance of the study in simple language and by referring to the help they will offer during the interview process. This can explain why our results showed that nationality was not associated with a lower F2F response.

Second, young people had higher nonresponse rates in our F2F survey than those in older age groups. This age effect was not found in the web study. Additional analysis showed that the higher F2F nonresponse was related only to their higher noncontact rates because refusal rates did not differ between different age groups. Previous studies also found higher noncontact rates among younger age groups [38,39]. This is attributed to the fact that this working-age population group is less likely to be at home when an interviewer contacts them than people older than 65 years, who are most often retired [38]. In a web survey, these interviewer contacts at home are not required, which may explain why no age effect was found in the web survey. Moreover, younger people have a high probability of meeting the necessary conditions (eg, internet access) and having the skills to participate in a web survey [5,9,35,36,40].

Third, people with low education levels are less likely to participate in surveys, regardless of the data collection modes used [6,13,20,23,41-43]. Our unit nonresponse analyses by mode system also confirmed this since lower educated people participated less in both studies. Nevertheless, this socioeconomic difference was stronger in the web survey than in the F2F survey. Reasons for this could be lower internet access rates [9,36,37] and less frequent internet use among low educated people [40]. Moreover, this socioeconomic group may have a greater need for interviewers to explain the importance of the survey and to motivate them to participate.

A considerable cost advantage of web versus F2F data collection was reported in this paper. Most other cost comparison studies have also found a major cost advantage when using the web versus other modes (F2F, telephone, paper). A cost comparison conducted in the framework of a cross-sectional parental survey on the mental health of children showed that web survey data collection (using postal mail recruitment and including one reminder letter) was 4 times cheaper than F2F data collection [6]. Substantial cost advantages (half of the cost) were also reported for web data collection (using mail invitations) compared to paper-and-pencil data collection in the context of a parental survey on children’s health status [22]. Sinclair et al [21] reported that a web survey (using postal mail invitations) offered a considerable cost advantage compared to a telephone survey organized in the context of a community-based health survey. Nevertheless, their paper-and-pencil survey using mail recruitment had lower costs than their web survey due to the high costs associated with web survey development. By using email instead of postal mail invitations, the cost advantage of a web survey over other modes would even be more pronounced. In most countries, emails cannot be sent to a random sample drawn from the general population because email addresses are not available for researchers to contact potential respondents. Denmark is an exception since a large proportion of Danish citizens have a mandatory digital mailbox that is used for communication with public authorities and to request survey participation [42].

### Conclusion

The use of F2F data collection should be preferred over the use of single-mode web data collection for population-based HISs. This recommendation is based on (1) the considerably lower unit response rates of the web survey compared to the F2F survey (18% vs 43%), (2) markedly lower response rates from some specific sociodemographic groups in the web survey versus F2F survey (ie, older people, low educated people, people of foreign nationalities, and people living alone), and (3) the nonresponse analyses which showed that certain sociodemographic groups—people with low education level, of non-Belgian nationalities, and living in the Brussels-Capital or Walloon regions—were more disadvantaged in a web study compared to an F2F study.

Lower response rates can induce more bias in measured HIS indicators because there is a greater chance that web respondents and nonrespondents show differences not only in terms of these sociodemographic characteristics but also in terms of their health status and health behavior characteristics. Moreover, if response rates show greater differences between various sociodemographic groups, this will affect comparisons between different sociodemographic groups. Single-mode web data collection would therefore better be restricted to specific target groups with universal internet access (eg, university students, online panel members) and should preferably not be used for surveys organized in the general population. This study did, however, show that web data collection offered a considerable cost advantage compared to F2F data collection.

### Recommendations and Future Prospects

In order to benefit from this cost advantage to some extent without increasing the risk of nonresponse bias, the web mode could be integrated in a mixed-mode design. This approach, in which some respondents complete the questionnaire on the web and other respondents (ie, those unwilling/incapable to participate online) use another mode, is already being tested and used in multiple European HISs [44-46]. A specific mixed-mode methodology, push-to-web, could, for example, be considered: people would first be invited by postal mail to participate online and they would then be contacted by an interviewer only in case of nonparticipation [47].

Based on the results of this study, experimenting with adaptive survey designs in which different sample members are assigned to different data collection modes can also be recommended. A potential strategy could be to invite only sociodemographic groups more eager to participate online for a web HIS while continuing to focus on increasing F2F response rates for the other sociodemographic groups. Tailoring the HIS data collection to different sociodemographic groups could reduce the nonresponse bias without increasing the costs.

When considering mode changes, it might also be useful to experiment with adapted recruitment procedures. Elements to take into account in future research could include the incentives for participation, the number and type of reminders, the use of tailored invitation letters based on age group, and the allocation of experienced interviewers to work with difficult to reach subpopulations.

When evaluating new designs, it might be worthwhile to focus not only on unit response rates but also on other less ambiguous indicators of nonresponse bias. These could include the calculation of R-indicators (R stands for representativeness), indicators that measure the similarity between the respondents to a survey and the sample or the population under investigation [48].

## Appendix

Multimedia Appendix 1 Sampling, sample substitution, recruitment procedure, and proxy interviewing.

Multimedia Appendix 2 Checklist for Reporting Results of Internet E-Surveys (CHERRIES).

## Abbreviations

BHIS: Belgian Health Interview Survey
BHISF2F: Belgian Health Interview Survey by F2F edition 2018
BHISWEB: Belgian Health Interview Survey by Web
CAPI: computer-assisted personal interview
EHIS: European Health Interview Survey
Eurostat: European Statistical Office
F2F: face-to-face
OR: odds ratio
Statbel: Belgian Statistical Office
