# Supplementary material for: Unit Response and Costs in Web Versus Face-To-Face Data Collection: Comparison of Two Cross-sectional Health Surveys
Source: J Med Internet Res. 2022 Jan 7;24(1):e26299. doi: 10.2196/26299 (PMC8783289; doi:10.2196/26299)
Supplement: Multimedia Appendix 1 [file jmir_v24i1e26299_app1.docx]

# Multimedia appendix 1: Sampling, sample substitution, recruitment procedure and proxy interviewing

## Belgian Health Interview Survey by face-to-face

Sampling

A multistage clustered sampling procedure was applied. The first step of this sampling strategy consisted of a regional and provincial stratification. In the second step, municipalities were selected in all strata according to a ‘probability proportional to size’ sampling approach. In the third step households were sampled within each municipality using a systematic sampling approach. The national register was used to draw the sample. For every household selected for the BHISF2F, three replacement households matched on statistical sector, household size and age of the reference person were as well selected. Besides this cluster of four households, a substitute cluster of another four households was created in case of non-participation of all first cluster households. This latter cluster had no shared characteristics with the initial household cluster.

Sample substitution

Sample substitution was applied during data collection: non-participating households were substituted, if needed several times, by replacement households.

Recruitment procedure

Prior to the contact attempts of an interviewer, the households selected for the BHISF2F received a postal advance letter that stated that an interviewer would come by and that contained information on the usefulness and voluntary character of the BHIS. If the household could not be contacted, refused to participate or if the interview was impossible, interviewers had to contact the replacement household. The status of ‘non-contactable’ could only be achieved after making at least five contact attempts which had to include at least one home visit and which had to occur at different days of the week and at different hours of the day. There was no material or monetary incentive for BHISF2F participation. In households with maximum four members, all members were selected for participation. For households with at least five members, only four members had to participate. These members were selected based on the following approach: the reference person and, when applicable, his or her partner were always selected and the other household members were selected using a random sampling approach.

Proxy interviewing

Proxy interviewing (another person, most often a household member, replying on behalf of the selected household member) was mandatory for selected individuals younger than 15 years and for those physically or mentally not capable to respond themselves. Moreover, it could be applied for selected individuals who were absent for a long time (at least one month) and for those who explicitly refused to participate themselves, but permitted a proxy interview.

## Belgian Health Interview Survey by Web

Sampling

Contrary to the BHISF2F, this study was organized on individual instead of household level and only individuals aged 16 till 85 could be selected. Moreover, individuals living in collective or institutional households and individuals living in the Germany speaking region of Belgium (East Belgium, <1% of the Belgian population) were excluded from the sampling frame. A multistage clustered sampling procedure, similar to the BHISF2F, was used to select individuals for the BHISWEB study. The first step of this sampling strategy consisted of a regional and provincial stratification. In the second step, municipalities were selected in all strata according to a ‘probability proportional to size’ sampling approach. In the third step individuals were sampled within each municipality using a systematic sampling approach. For every selected individual, nine replacement individuals comparable in terms of statistical sector (i.e., a subdivision of municipality), sex and age were selected too.

Sample substitution

Matched sample substitution was applied during data collection: non-respondents were replaced, if needed several times, by replacement individuals.

Recruitment

Selected individuals were invited through a postal letter since no sampling frame of e-mail addresses of the general population is available in Belgium. This invitation letter included a hyperlink and login details to access the questionnaire and covered information on the purpose, the content and the voluntary character of the study. Furthermore, it stated that the questionnaire had to be completed during the 14 days’ access period and that participants would receive a €10 conditional incentive in the form of a gift voucher. This voucher could be spent in different types of stores, including supermarkets, do-it-yourself stores and clothes shops. The reminder procedure consisted of sending one reminder letter to non-respondents seven days after the invitation letter.

Proxy interviewing

Proxy interviewing was not allowed.
