# Supplementary material for: Unit Response and Costs in Web Versus Face-To-Face Data Collection: Comparison of Two Cross-sectional Health Surveys
Source: J Med Internet Res. 2022 Jan 7;24(1):e26299. doi: 10.2196/26299 (PMC8783289; doi:10.2196/26299)
Supplement: Multimedia Appendix 2 [file jmir_v24i1e26299_app2.docx]

# **Checklist for Reporting Results of Internet E-Surveys (CHERRIES)**

| **Item Category** | **Checklist Item** | **Explanation** |
| --- | --- | --- |
| **Design** | Describe survey design | Target population: The Belgian population aged 16 till 85 years old, excluding individuals living in institutionalized households or East-Belgium.  Sample frame: The Belgian National Register |
| **IRB (Institutional Review Board) approval and informed consent process** | IRB approval | Approval was received from the Belgian Privacy commission and from the Ethics Committee of the Ghent University Hospital. |
|  | Informed consent | Selected individuals received an invitation letter including a folder with additional information. Through these materials and the welcome screen of the web questionnaire potential participants were informed about the length of the survey, the principal investigator, the purpose of the study, the confidentiality of the data, etc. Before being able to start the survey completion, respondents had to indicate that they agreed with the terms and conditions of the study. |
|  | Data protection | The address data of the selected individuals were only stored at our fieldwork partner. This assured a separation of the collected health data and the personal identifying data. All data stored at Sciensano is stored on secure servers and access is limited to scientists who have an interest in the study. |
| **Development and** **re-testing** | Development and testing | The questionnaire items derived from the European Health Interview Survey, wave 3. Before the launch, the web questionnaire was tested by questionnaire experts and a convenience sample of acquaintances. |
| **Recruitment process and description of the sample having access to the questionnaire** | Open survey versus closed survey | A closed survey: only individuals receiving a personal invitation with a username and password could participate (password-protected). |
|  | Contact mode | One postal invitation letter and one postal reminder letter sent after one week. |
|  | Advertising the survey | Not applicable since only postal invitations were sent. |
| **Survey administration** | Web/E-mail | A web survey |
|  | Context | No publication on a website, selected individuals received a direct link to the questionnaire. |
|  | Mandatory/voluntary | The survey was completely voluntary. |
|  | Incentives | People fully completing the questionnaire received of voucher of 10 euros that could be spent in different types of stores. |
|  | Time/Date | The survey was available online from April till November 2018, with a break during the holiday months. |
|  | Randomization of items or questionnaires | No randomization or alternation of questionnaire items or questionnaires was applied. |
|  | Adaptive questioning | To reduce the number and complexity of the questionnaire adaptive questioning was applied. |
|  | Number of Items | The mean number of items per screen was 1.6 and the total number of items was 195 of which 70 were conditional to previous responses. |
|  | Number of screens (pages) | The total number of screens depended on the answers given. |
|  | Completeness check | A non-response option was foreseen for every question (“I don’t know” and “I prefer not to say”). When respondents did not answer the questions they received a warning message before being able to continue with the questionnaire. No consistency of completeness checks were done before the questionnaire was submitted. |
|  | Review step | A “back” button was provided such that participants could change a previously given answer. A review step which displays a summary of the responses and asks the respondents if they are correct was not included. |
| **Response rates** | Unique site visitor | This was not foreseen. |
|  | View rate (Ratio of unique survey visitors/unique site visitors) | We were not able to calculate the view rate. |
|  | Participation rate (Ratio of unique visitors who agreed to participate/unique first survey page visitors) | In our study, the response rate was calculated as follows: the individuals having completed the first questions of the three first modules (i.e. a set of questions related to the same topic) of the web questionnaire/ the total amount of invited individuals. The (unweighted) response rate: 16.3 % (1010/6183). |
|  | Completion rate (Ratio of users who finished the survey/users who agreed to participate) | Among the 1032 people who agreed to participate 48 broke of the survey before reaching the end (4.7%). |
| **Preventing** multiple  entries from  the same  individual | Cookies used | Authentication cookies were not used. |
|  | IP check | IP addresses were not collected. |
|  | Log file analysis | Log file analysis were not used to assess multiple entries. |
|  | Registration | Duplicate data entry was controlled by the fact that a username and password was needed to access the survey. Participants were able to access and make changes to their completed questionnaire for as long as their access period. If changes were made to the first data entry, the last version was kept for the analyses. |
| **Analysis** | Handling of incomplete questionnaires | In our further studies, we only assessed the answers of participants replying to the first questions of the three first modules of the questionnaire. |
|  | Questionnaires submitted with an atypical timestamp | We measured the time stamp at the beginning and ending of the questionnaire completion. Completing the whole questionnaire took on average 30 minutes (range 11-90 minutes). People with atypical timestamps were not by default excluded. |
|  | Statistical correction | In this study, weighting methods were applied to adjust for the differences between the gross sample distribution of both studies. |

Eysenbach G. Improving the Quality of Web Surveys: The Checklist for Reporting Results of Internet E-Surveys (CHERRIES). J Med Internet Res [Internet] 2004 Sep 29 [cited 2021 Mar 23];6(3). PMID:15471760
